# Supplementary material for: Ensuring robustness in scientific research, split-root assays as an example case
Source: Quant Plant Biol. 2025 Aug 13;6:e25. doi: 10.1017/qpb.2025.10004 (PMC12451248; doi:10.1017/qpb.2025.10004)
Supplement: Salvatore et al. supplementary material [file S2632882825100040sup001.docx]

# Supplementary Materials

Supplementary Figure 1: **Comparison of the LRs numbers of Arabidopsis split-root for different nitrogen availabilities in a short (15 DAG) and a long (20 DAG) protocol.** The Number of LR of *Arabidopsis thaliana* was measured in response to 5 days of treatment in split-root conditions in either 15 days old seedlings (left panel) or in 20 days old seedlings (right panel). Seedlings were grown in d-root systems the entire treatment and the cut was performed with a razor blade. LRs responses to different conditions: the high nitrogen (10mM KNO3) in both sides of the split-root condition, HNHN; the side of high nitrogen in the split-root heterogeneous condition, HNln; the side of low nitrogen in the split-root heterogeneous condition, LNhn; and the low nitrogen (0.2mM KNO3) in both sides of the split-root condition, LNLN. Boxplot displays the median of each group bounded by the first and third quartile. Asterisks indicate the significance in the comparison of two nitrogen treatments: *P<0.05; **P<0.01; ***P<0.001. The HNln vs LNhn split-root treatment was compared using a Wilcoxon test and the rest of the comparisons were performed using a Mann-Whitney test.


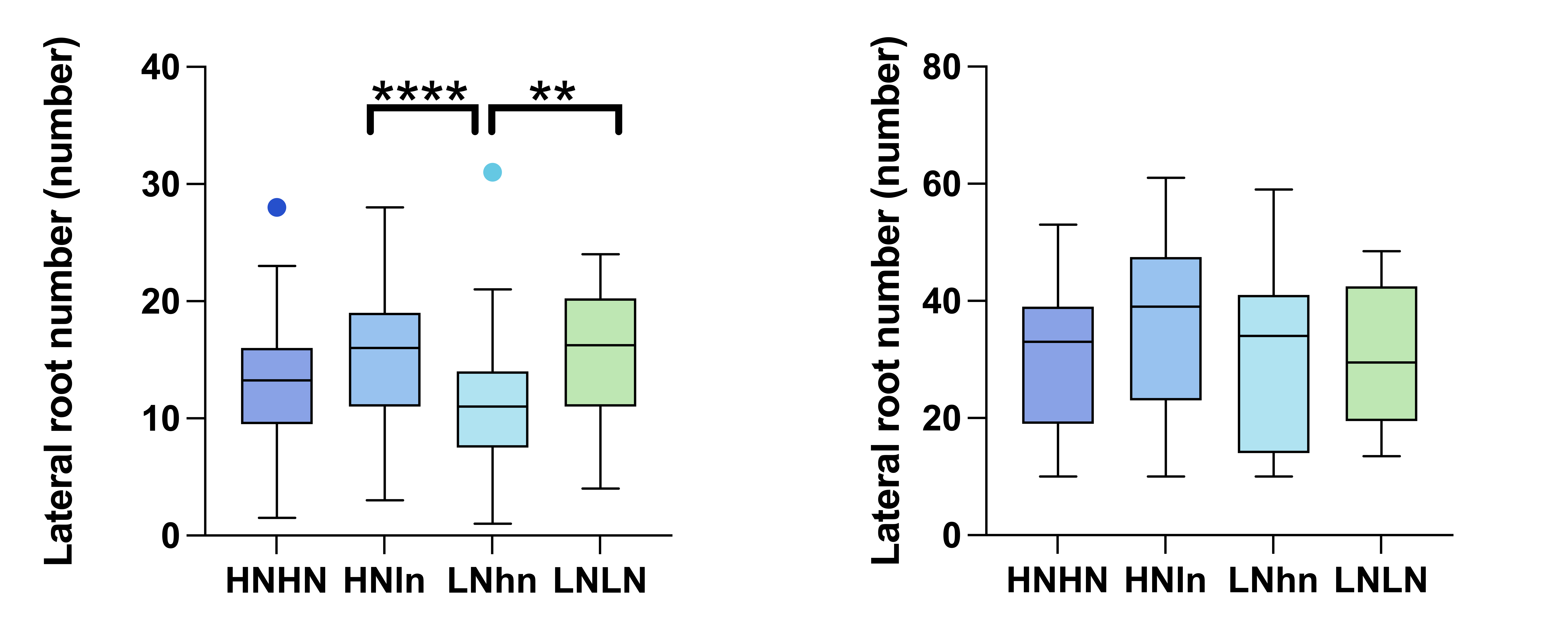


Supplementary Figure 2: **Diagram of the different sections in the root analyses.** Blue lines indicate the position of the root at the start of split root treatment and are routinely used during experiments. The old part represents the section of the main root of the plant that had already developed at day 0 of the treatment when the plant was transferred to the split-root plate, and its associated LRs. The new part represents the section of the main root that developed during the treatment and its associated LRs. The total part of the roots is the sum of the old and new parts.


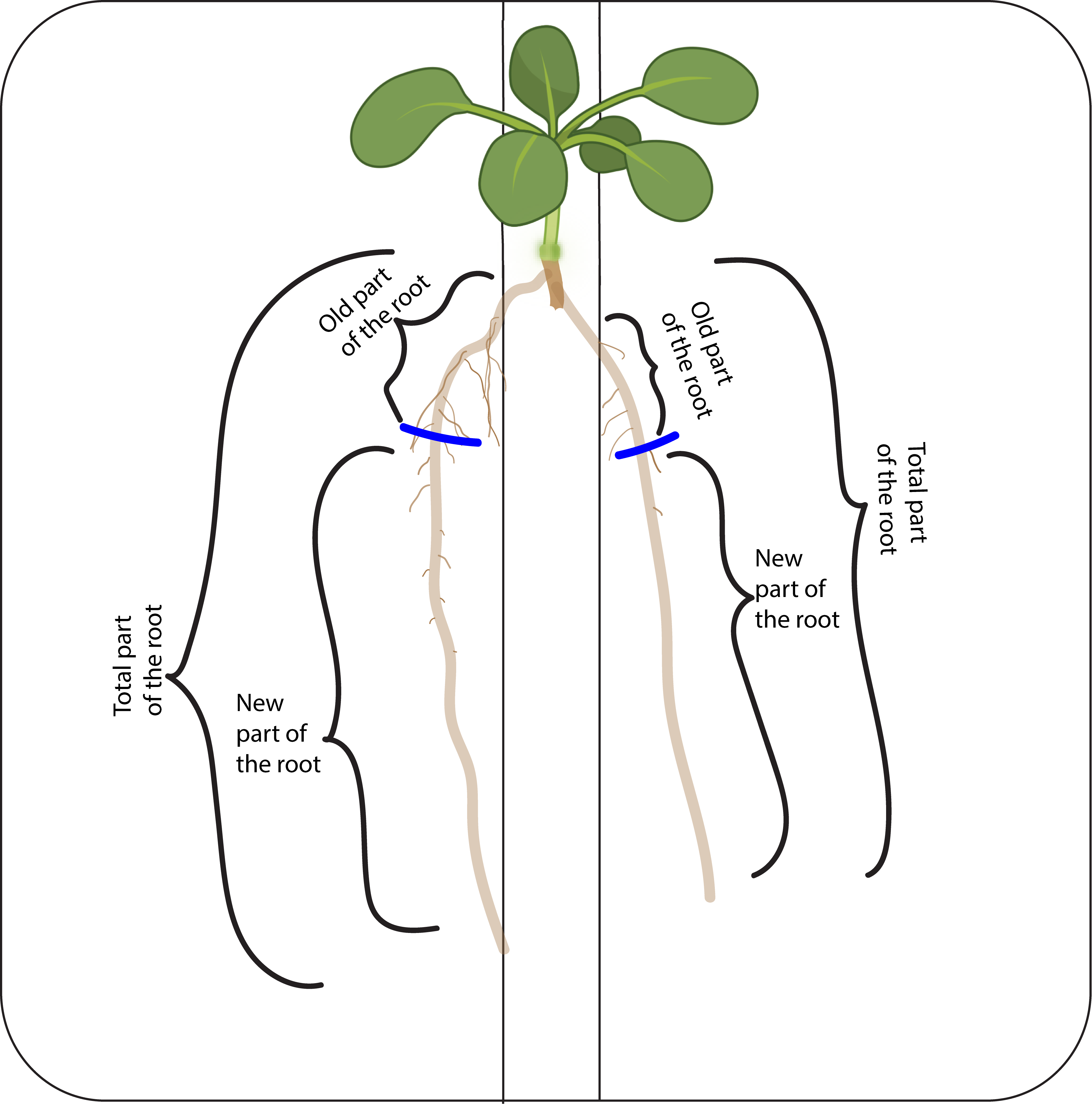


Supplementary Figure 3: **Comparison of the LR density of Arabidopsis in split-root for different nitrogen availability in different sections of the root.** *Arabidopsis thaliana* root system response in 15 DAG old seedlings after 5 days of treatment in split-root conditions. Seedlings were grown in d-root systems the entire treatment and the cut was performed with a razor blade. LR density in response to each condition, high nitrogen (10mM KNO3) in both sides of the split-root condition (HNHN), the side of high nitrogen in the split-root heterogeneous condition (HNln), the side of low nitrogen in the split-root heterogeneous condition (LNhn), and the low nitrogen (0.2mM KNO3) in both sides of the split-root condition (LNLN). The LR density was measured for 3 sections, the new section (left panel), the old section (middle panel), and the total section (right panel). Asterisks indicate the significance in the comparison of two nitrogen treatments: *P<0.05; **P<0.01; ***P<0.001. The HNln vs LNhn split-root treatment was compared using a Wilcoxon test and the rest of the comparisons were performed using a Mann-Whitney test.


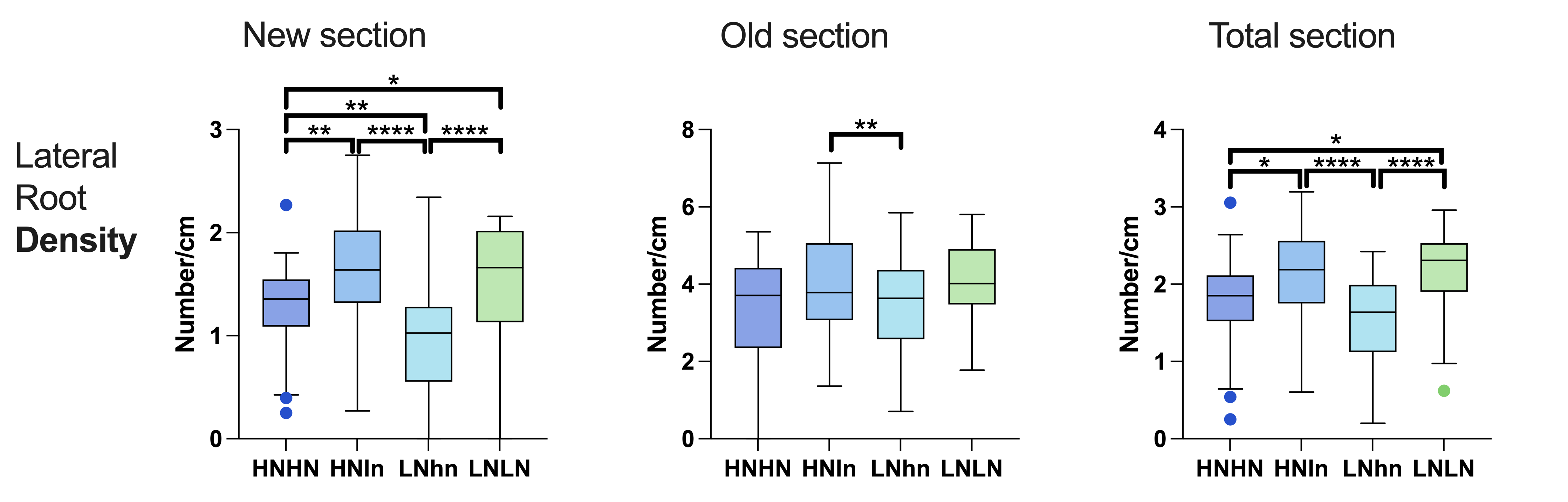


Supplementary Figure 4: **Comparison of the LR density of Arabidopsis in split-root for different nitrogen availability with the roots covered or exposed to light.** *Arabidopsis thaliana* 15DAG old root system response after 5 days of treatment in split-root conditions in either seedlings with roots exposed at light (non-covered) (left) or seedlings with roots under D-root system (right). Seedlings were cut with a razor blade when the protocol was performed. LRs responses to each condition, high nitrogen (10mM KNO3) in both sides of the split-root condition (HNHN), the side of high nitrogen in the split-root heterogeneous condition (HNln), the side of low nitrogen in the split-root heterogeneous condition (LNhn), and the low nitrogen (0.2mM KNO3) in both sides of the split-root condition (LNLN). Asterisks indicate the significance in the comparison of two nitrogen treatments: *P<0.05; **P<0.01; ***P<0.001. The HNln vs LNhn split-root treatment was compared using a Wilcoxon test and the rest of the comparisons were performed using a Mann-Whitney test.


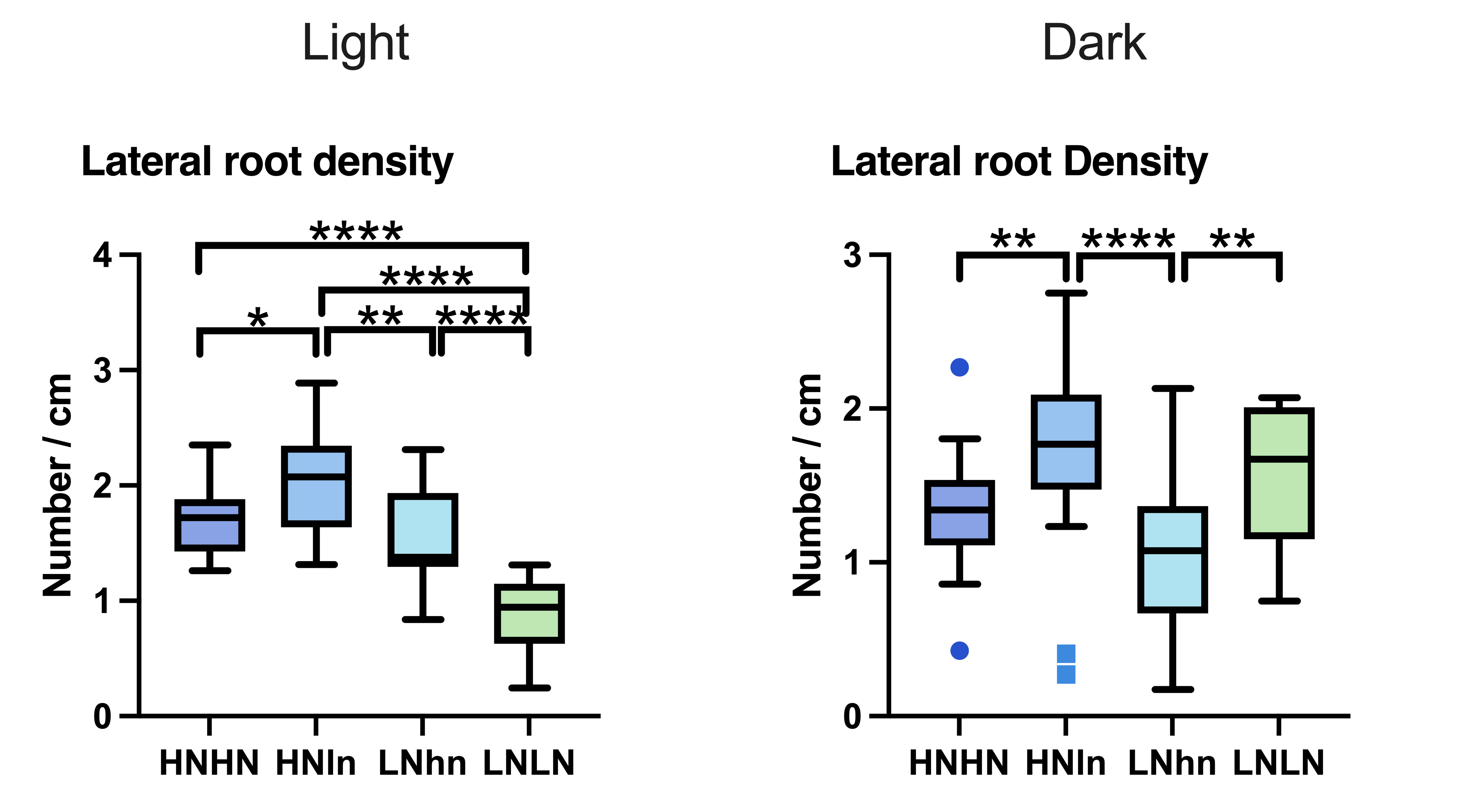


Supplementary Figure 5: **Representative images of A. thaliana seedlings after each treatment.** *Arabidopsis thaliana* 15DAG root system response after 5 days of treatment in split-root conditions in either seedlings with roots exposed to light (non-covered) (A, C, E) or seedlings with roots grown using the D-root system (B, D, F). Seedling responses are shown to the different nitrate treatments, LNLN (A, B), HNHN (C, D), and HNln + LNhn (E, F).


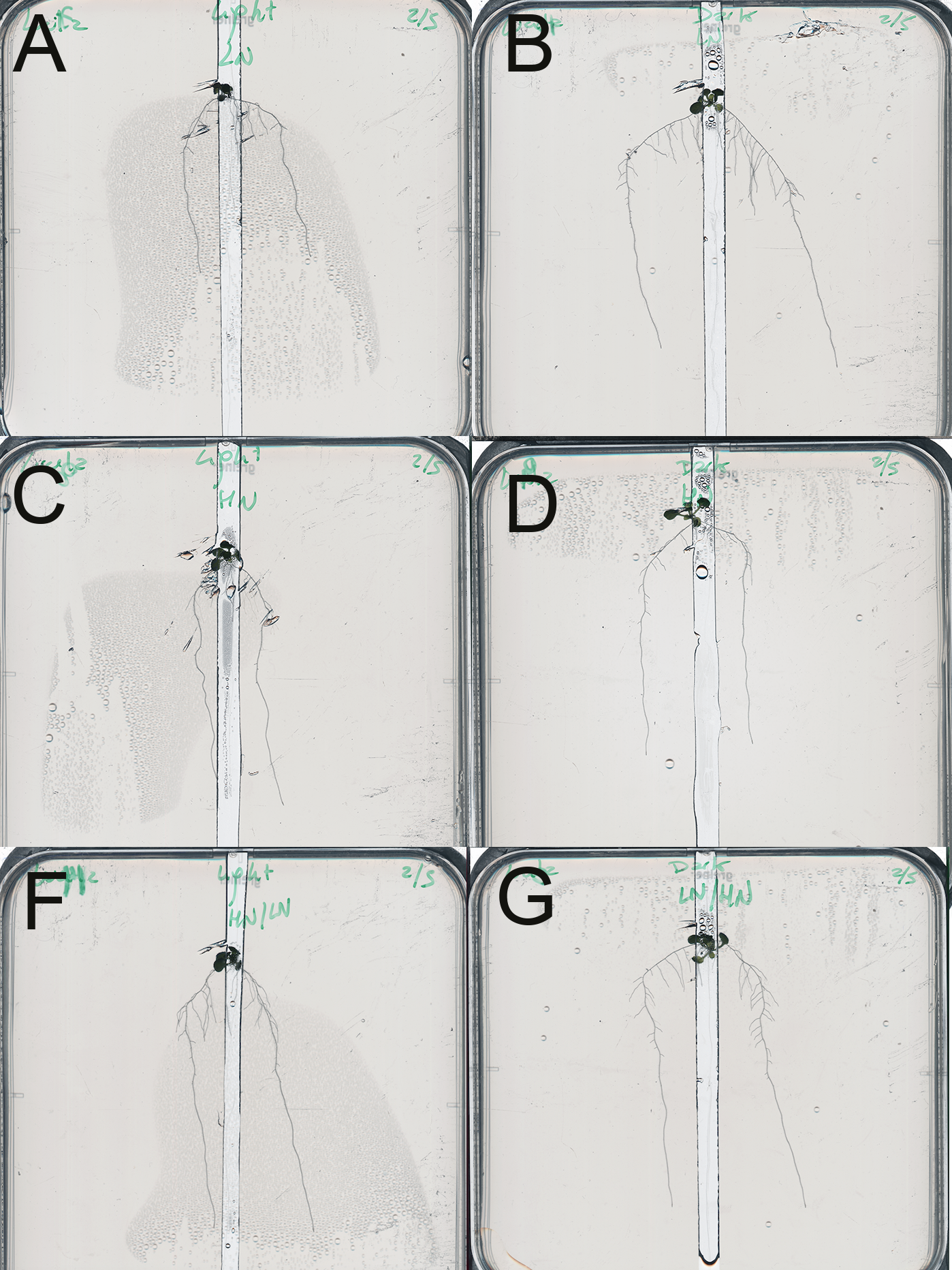


**Table S1. P-Values for each comparison made in Figure 3.** From Left to Right – Top to Bottom. A. Table for the comparison made for the summed lateral root length for the 15 DAG protocol. B. Table for the comparison made for the summed lateral root length for the 20 DAG protocol. C. Table for the comparison made for the average lateral root length for the 15 DAG protocol. D. Table for the comparison made for the average lateral root length for the 20 DAG protocol. Statistics performed for these experiments are better detailed in the Figures they are referring. Significance in between the pair-comparison is visualized with a green background.

**Table S2. P-Values for each comparison made in Figure 4.** From Left to Right – Top to Bottom. A. Table for the comparison made for the summed lateral root length for the New section. B. Table for the comparison made for the summed lateral root length for the Old section. C. Table for the comparison made for the summed lateral root length for the Total section. D. Table for the comparison made for the average lateral root length for the New section. E. Table for the comparison made for the average lateral root length for the Old section. F. Table for the comparison made for the average lateral root length for the Total section. G. Table for the comparison made for the lateral root number for the New section. H. Table for the comparison made for the lateral root number for the Old section. I. Table for the comparison made for the lateral root number for the Total section Statistics performed for these experiments are better detailed in the Figures they are referring. Significance in between the pair-comparison is visualized with a green background.

**Table S3. P-Values for each comparison made in Figure 5.** From Left to Right – Top to Bottom. A. Table for the comparison made for the summed lateral root length for the light protocol. B. Table for the comparison made for the summed lateral root length for the dark protocol. C. Table for the comparison made for the lateral root number for the light protocol. D. Table for the comparison made for the lateral root number for dark protocol. Statistics performed for these experiments are better detailed in the Figures they are referring. Significance in between the pair-comparison is visualized with a green background.

**Table S4. P-Values for each comparison made in Figure S1.** From Left to Right – Top to Bottom. A. Table for the comparison made for the lateral root number for the 15 DAG protocol. B. Table for the comparison made for the lateral root number for the 20 DAG protocol. Statistics performed for these experiments are better detailed in the Figures they are referring. Significance in between the pair-comparison is visualized with a green background.

**Table S5. P-Values for each comparison made in Figure S3.** From Left to Right – Top to Bottom. A. Table for the comparison made for the lateral root density for the New section of the root. B. Table for the comparison made for the lateral root density for the Old section of the root. C. Table for the comparison made for the lateral root density for the total section of the root. Statistics performed for these experiments are better detailed in the Figures they are referring. Significance in between the pair-comparison is visualized with a green background.

**Table S6. P-Values for each comparison made in Figure S4.** From Left to Right – Top to Bottom. A. Table for the comparison made for the lateral root density for the light protocol. B. Table for the comparison made for the lateral root density for the dark protocol. Statistics performed for these experiments are better detailed in the Figures they are referring. Significance in between the pair-comparison is visualized with a green background.

Supplementary File 1: **Protocol to achieve an efficient split-root assay**

# Supplementary material

**Table S1. P-Values for each comparison made in Figure 3.**

A B

| Comparison | HNHN | HNln | LNhn | LNLN |
| --- | --- | --- | --- | --- |
| HNHN |  | 0.0497 | 0.3679 | 0.0279 |
| HNln |  |  | <0.0001 | 0.5086 |
| LNhn |  |  |  | 0.0008 |
| LNLN |  |  |  |  |

| Comparison | HNHN | HNln | LNhn | LNLN |
| --- | --- | --- | --- | --- |
| HNHN |  | 0.01 | 0.3699 | 0.0186 |
| HNln |  |  | 0.0007 | 0.682 |
| LNhn |  |  |  | 0.2022 |
| LNLN |  |  |  |  |

C D

| Comparison | HNHN | HNln | LNhn | LNLN |
| --- | --- | --- | --- | --- |
| HNHN |  | 0.0003 | 0.0365 | <0.0001 |
| HNln |  |  | 0.0505 | 0.5761 |
| LNhn |  |  |  | 0.0486 |
| LNLN |  |  |  |  |

| Comparison | HNHN | HNln | LNhn | LNLN |
| --- | --- | --- | --- | --- |
| HNHN |  | 0.0162 | 0.6864 | 0.0028 |
| HNln |  |  | 0.0112 | 0.2141 |
| LNhn |  |  |  | 0.0035 |
| LNLN |  |  |  |  |

**Table S2. P-Values for each comparison made in Figure 4**

A B C

| Comp | HNHN | HNln | LNhn | LNLN |
| --- | --- | --- | --- | --- |
| HNHN |  | 0.0497 | 0.3679 | 0.0279 |
| HNln |  |  | <0.0001 | 0.5086 |
| LNhn |  |  |  | 0.0008 |
| LNLN |  |  |  |  |

| Comp | HNHN | HNln | LNhn | LNLN |
| --- | --- | --- | --- | --- |
| HNHN |  | 0.1428 | 0.4505 | 0.0110 |
| HNln |  |  | <0.0001 | 0.1457 |
| LNhn |  |  |  | 0.0002 |
| LNLN |  |  |  |  |

| Comp | HNHN | HNln | LNhn | LNLN |
| --- | --- | --- | --- | --- |
| HNHN |  | 0.0032 | 0.1908 | 0.0979 |
| HNln |  |  | <0.0001 | 0.3101 |
| LNhn |  |  |  | 0.0141 |
| LNLN |  |  |  |  |

| Comp | HNHN | HNln | LNhn | LNLN |
| --- | --- | --- | --- | --- |
| HNHN |  | 0.0093 | 0.2078 | 0.7148 |
| HNln |  |  | 0.0282 | 0.035 |
| LNhn |  |  |  | 0.5424 |
| LNLN |  |  |  |  |

| Comp | HNHN | HNln | LNhn | LNLN |
| --- | --- | --- | --- | --- |
| HNHN |  | 0.0038 | 0.1368 | 0.0003 |
| HNln |  |  | <0.0001 | 0.1981 |
| LNhn |  |  |  | <0.0001 |
| LNLN |  |  |  |  |

| Comp | HNHN | HNln | LNhn | LNLN |
| --- | --- | --- | --- | --- |
| HNHN |  | 0.0078 | 0.5587 | 0.0018 |
| HNln |  |  | 0.0112 | 0.2141 |
| LNhn |  |  |  | 0.0035 |
| LNLN |  |  |  |  |

D E F

| Comp | HNHN | HNln | LNhn | LNLN |
| --- | --- | --- | --- | --- |
| HNHN |  | 0.0645 | 0.1633 | 0.1328 |
| HNln |  |  | <0.0001 | >0.99 |
| LNhn |  |  |  | 0.004 |
| LNLN |  |  |  |  |

G H I

| Comp | HNHN | HNln | LNhn | LNLN |
| --- | --- | --- | --- | --- |
| HNHN |  | 0.0111 | 0.0080 | 0.2090 |
| HNln |  |  | <0.0001 | 0.2638 |
| LNhn |  |  |  | 0.0006 |
| LNLN |  |  |  |  |

| Comp | HNHN | HNln | LNhn | LNLN |
| --- | --- | --- | --- | --- |
| HNHN |  | 0.5939 | 0.5337 | 0.1931 |
| HNln |  |  | 0.0689 | 0.2292 |
| LNhn |  |  |  | 0.0557 |
| LNLN |  |  |  |  |

**Table S3. P-Values for each comparison made in Figure 5.**

| Comparison | HNHN | HNln | LNhn | LNLN |
| --- | --- | --- | --- | --- |
| HNHN |  | 0.0074 | 0.7707 | 0.1736 |
| HNln |  |  | 0.0006 | 0.2488 |
| LNhn |  |  |  | 0.3457 |
| LNLN |  |  |  |  |

A B

| Comparison | HNHN | HNln | LNhn | LNLN |
| --- | --- | --- | --- | --- |
| HNHN |  | 0.0111 | 0.0948 | <0.0001 |
| HNln |  |  | 0.0053 | <0.0001 |
| LNhn |  |  |  | <0.0001 |
| LNLN |  |  |  |  |

| Comparison | HNHN | HNln | LNhn | LNLN |
| --- | --- | --- | --- | --- |
| HNHN |  | 0.0092 | 0.2153 | 0.4277 |
| HNln |  |  | <0.0001 | 0.1135 |
| LNhn |  |  |  | 0.0466 |
| LNLN |  |  |  |  |

| Comparison | HNHN | HNln | LNhn | LNLN |
| --- | --- | --- | --- | --- |
| HNHN |  | 0.029 | 0.0409 | <0.0001 |
| HNln |  |  | 0.0006 | <0.0001 |
| LNhn |  |  |  | <0.0001 |
| LNLN |  |  |  |  |

C D

**Table S4. P-Values for each comparison made in Figure S1.**

| Comparison | HNHN | HNln | LNhn | LNLN |
| --- | --- | --- | --- | --- |
| HNHN |  | 0.00645 | 0.1633 | 0.1328 |
| HNln |  |  | <0.0001 | 0.9979 |
| LNhn |  |  |  | 0.0040 |
| LNLN |  |  |  |  |

| Comparison | HNHN | HNln | LNhn | LNLN |
| --- | --- | --- | --- | --- |
| HNHN |  | 0.2732 | 0.8742 | 0.9105 |
| HNln |  |  | 0.1628 | 0.3257 |
| LNhn |  |  |  | 0.8889 |
| LNLN |  |  |  |  |

A B

**Table S5. P-Values for each comparison made in Figure S3.**

A B C

| Comp | HNHN | HNln | LNhn | LNLN |
| --- | --- | --- | --- | --- |
| HNHN |  | 0.1457 | 0.8950 | 0.1057 |
| HNln |  |  | 0.0033 | 0.7111 |
| LNhn |  |  |  | 0.0591 |
| LNLN |  |  |  |  |

| Comp | HNHN | HNln | LNhn | LNLN |
| --- | --- | --- | --- | --- |
| HNHN |  | 0.0163 | 0.0620 | 0.0130 |
| HNln |  |  | <0.0001 | 0.7580 |
| LNhn |  |  |  | <0.0001 |
| LNLN |  |  |  |  |

|  | HNHN | HNln | LNhn | LNLN |
| --- | --- | --- | --- | --- |
| HNHN |  | 0.0020 | 0.0028 | 0.0370 |
| HNln |  |  | <0.0001 | 0.6207 |
| LNhn |  |  |  | <0.0001 |
| LNLN |  |  |  |  |

**Table S6. P-Values for each comparison made in Figure S4.**

A B

| Comparison | HNHN | HNln | LNhn | LNLN |
| --- | --- | --- | --- | --- |
| HNHN |  | 0.0240 | 0.1247 | <0.0001 |
| HNln |  |  | 0.0046 | <0.0001 |
| LNhn |  |  |  | <0.0001 |
| LNLN |  |  |  |  |

| Comparison | HNHN | HNln | LNhn | LNLN |
| --- | --- | --- | --- | --- |
| HNHN |  | 0.0033 | 0.0920 | 0.1873 |
| HNln |  |  | <0.0001 | 0.2946 |
| LNhn |  |  |  | 0.0052 |
| LNLN |  |  |  |  |
